# Design, evaluation and application of a modular 32 channel transmit/receive surface coil array for cardiac MRI at 7T

**DOI:** 10.1186/1532-429X-15-S1-W2

**Published:** 2013-01-30

**Authors:** A Graessl, W Renz, F Hezel, H Pfeiffer, W Hoffmann, P Kellman, T Niendorf

**Affiliations:** 1 Berlin Ultrahigh Field Facility, Max-Delbrueck Center for Molecular Medicine, Berlin, Germany; 2 Siemens Healthcare, Erlangen, Germany; 3 Physikalisch-Technische Bundesanstalt (PTB), Berlin, Germany; 4 Laboratory of Cardiac Energetics, National Institute of Health/NHLBI, Bethesda, MD, USA; 5 Experimental and Clinical Research Center (ECRC), Charité Campus Buch, Humboldt-University, Berlin, Germany

## Background

Cardiac MR (CMR) at ultrahigh fields (≥7.0 T) is challenged by B1+ inhomogeneities and signal voids which bear the potential to spoil the signal-to-noise (SNR) benefits at 7.0 T. There is a trend towards an ever larger number of transmit and receive channels, to support multi-dimensional B1+ shimming and parallel imaging. Therefore this work proposes a modular, two dimensional 32-channel transmit and receive array using loop elements and examines its efficacy for enhanced B1+ homogeneity and improved parallel imaging performance.

## Methods

**Figure 1:**
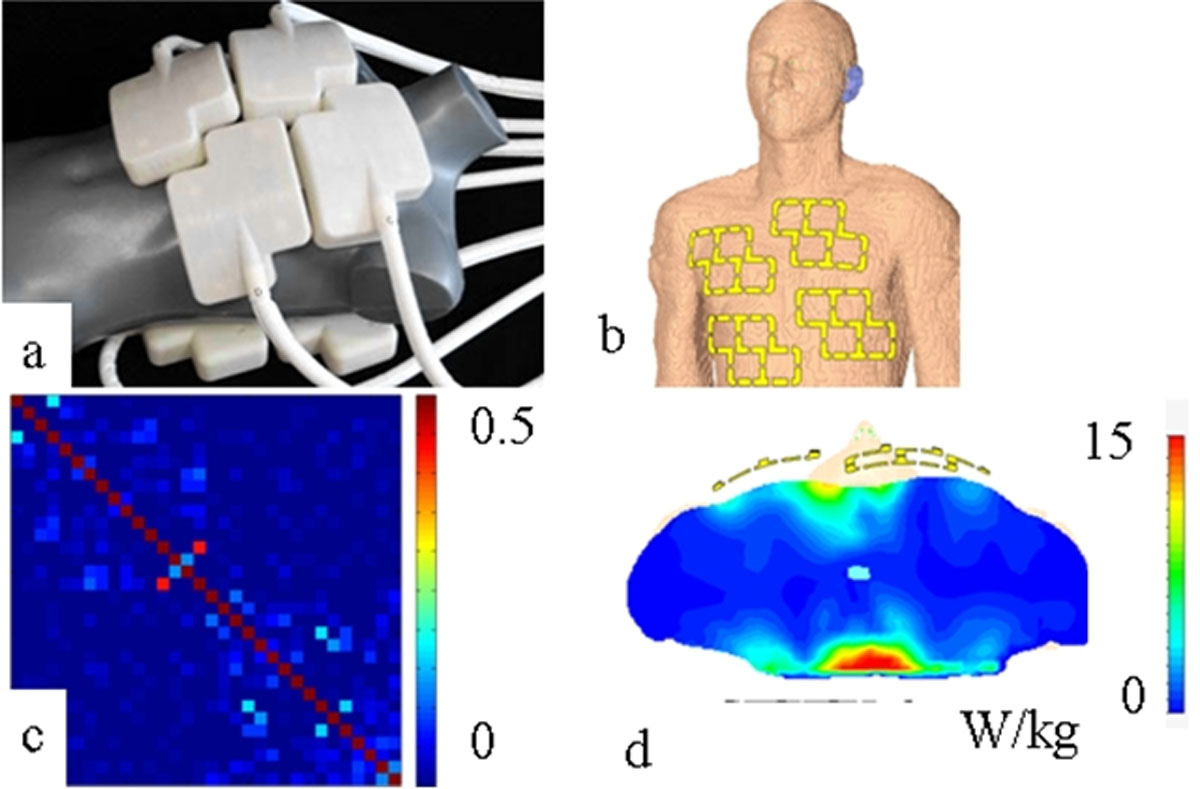
a) The coil placed on a mannequin. b) Basic scheme of the coil design and positioning on the anterior chest. c) SAR distribution derived from EMF simulations for transversal slice including the overall SAR maximum. d) 32 x 32 noise correlation matrix obtained for the proposed coil design.

The coil array consists of eight modules. Each module comprises 4 independent transceiver loop elements with a rectangular size of 6 cm x 6 cm each (Fig. 1a, b). Adjacent elements share a common conductor with an adjustable capacitor for decoupling of neighboring elements. Inter module decoupling is suppressed by a distance of 3 cm between neighboring loop structures. The MR experiments were conducted on a 7 T scanner (Siemens Healthcare, Erlangen, Germany). The amplifier output was split into 32 equal-intensity signals by means of home-built power splitters. Phase adjustments were implemented by phase-shifting coaxial . Electro-magnetic (EM) field and SAR simulations were performed using CST Studio Suite 2011 (CST AG, Darmstadt, Germany) together with voxel models from the Virtual Family. RF measurements and Cardiac MR using single breath-hold 2D CINE FLASH were done on three different subjects without subject-specific tuning and matching.

## Results

**Figure 2:**
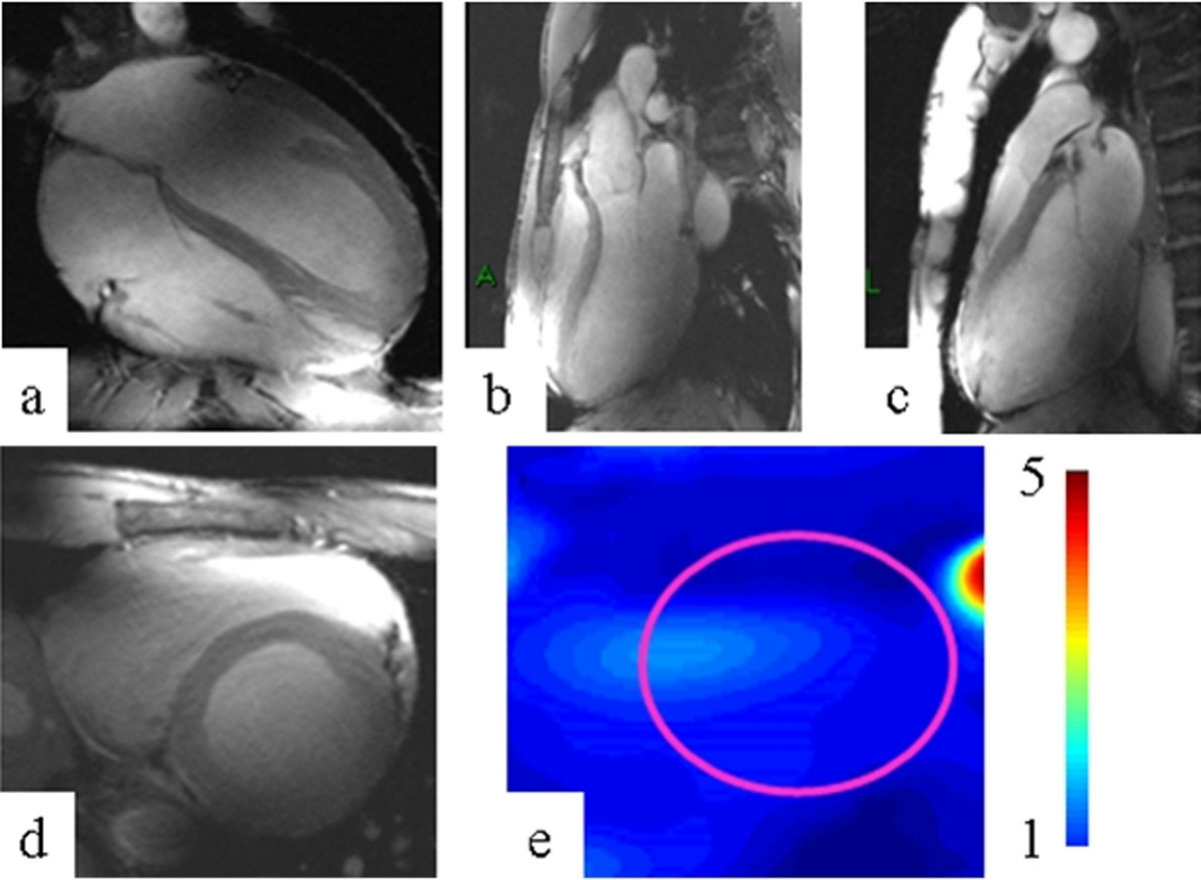
a-d) Images of the heart derived from 2D CINE FLASH imaging using the proposed coil design (voxel size 1x1x4 mm3, TE/TR=2.65/5.6 ms, BW=454 Hz): 4 chamber view, 3-chamber view, 2-chamber view, short axis view(R=2). e) GRAPPA g-factor map for short axis view and R=4.

The modules are lightweight (m< 400 g) and conform to a broad range of upper torso geometries. Reflection coefficients of -21± 7 dB were observed. Element coupling was below -10 dB for all elements and subjects. Noise correlation was 0.46 or well below for all elements and subjects (Fig. 1c). SAR values derived from EM simulations were well below the limits permitted by the IEC guidelines for an average power of 30 W (Fig. 1d). The overall image quality and the high spatial resolution of (1x1x4) mm3 enabled the visualization of subtle anatomic details (Fig. 2a-d).

## Conclusions

Our results demonstrate that the modular 32 channel coil array supports the acquisition of rather uniform images of the heart at 7.0 T. Combining a large number of surface coil elements yielded an excellent SNR and facilitated the depiction of subtle anatomical cardiac details. The parallel imaging performance enabled the acquisition of high resolution images with satisfactory SNR within one breath-hold (Fig. 2).

